# A tunable flat terahertz lens using Dirac semimetals: a simulation study

**DOI:** 10.1038/s41598-024-56026-0

**Published:** 2024-03-02

**Authors:** P. Panahianfar, B. Rezaei, A. Darafsheh

**Affiliations:** 1 Department of Condensed Matter Physics, Faculty of Physics, University of Tabriz, Tabriz, Iran; 2 Department of Radiation Oncology, Washington University School of Medicine in St. Louis, St. Louis, MO 63110 USA

**Keywords:** Photonic crystal, Graded index, Lens, Bulk Dirac semimetal, Terahertz, Optics and photonics, Applied optics, Microwave photonics

## Abstract

We propose and design a flat and tunable terahertz lens achieved through a two-dimensional photonic crystal composed of an array of rods made of a Dirac semimetal placed in air as the background medium. The structure of interest is a graded index photonic crystal, made possible by the slight variations in the rods’ radii in a direction perpendicular to the direction of the light propagation. Dirac semimetals' ability to respond to variations in their Fermi energy level manifested as a change in the refractive index provides the tunability of our proposed lens. The interaction of electromagnetic waves with the designed structure is investigated for both transverse magnetic and transverse electric polarizations using two-dimensional finite-difference time-domain method.

## Introduction

Photonic crystals (PCs) are dielectric structures with refractive index periodicity along one, two, or three directions of space^1–3^. Wavelength scale of the periodicity leads to formation of photonic bandgaps allowing light transport at permitted bands^4^. The photonic bandgaps and allowed bands can be exploited for application in optical waveguides^5^, cavities^6^, filters^1^, as well as for dynamically switchable devices^7–11^, and achieving self-collimation^7,12^ and negative refractive index^8,13^. Optical properties of PCs can be further tailored by introducing certain types of structural modifications along particular directions forming graded PCs (GPCs) whose parameters including refractive index^14^, filling factor of the unit cells^14^, and lattice period^15^, are gradually modified selectively. When the refractive index is modulated in GPCs, they are called graded-index (GRIN) PCs^16,17^. Such structures have attracted intense research interest for novel applications in light focusing^18–26^ and transport^27–29^, efficient mode coupling^30,31^, mode-order converting^32^, beam splitting^33^, wavelength demultiplexing^34^, and sensing^35^ including one-dimensional GRIN PC sensors composed of linearly^36–38^ and exponentially^39^ graded index layers. The graded index concept can also be exploited to engineer “super-narrow” photonic nanojets (PNJs) for a myriad of applications^40–43^ (a PNJ is a tightly focused light beam generated by transparent meso-scale objects^44–52^).

Terahertz (THz) spectral range has attracted intense attention due to a broad range of potential applications in medical and material sciences, as well as in homeland security and pharmaceutical industry^53,54^, many of which require dedicated focusing elements. Focusing properties of a lens are determined by its geometry and refractive index. Typical lenses have a fixed focal point at a given wavelength. Tunable lenses are lenses whose characteristics can be tuned non-destructively^55^. Electro-optical properties can be exploited to achieve a tunable lens^55^, in particular in PCs^56^ with applications in lasers^57^, optical switching^58^, and imaging^59^. GRIN PCs can be exploited not only to design a flat lens^60–64^, but also to design a dynamically tunable lens utilizing liquid crystals^56,65,66^, dielectric elastomers^67^, and semiconductors^68^. Although these methods provide dynamic control of a PC lens, they come with their own drawbacks. Dielectric elastomers’ response time is on the order of 100 ms^69^. Semiconductors have a limited operational bandwidth. The performance of liquid crystals, being anisotropic materials, is susceptible to temperature changes^70^; also, their response time to an applied external voltage is on the order of ms^71^, against the ps-ns time scale for Dirac semimetals^72,73^.

Dirac semimetals (DSs) have attracted research interests for potential light manipulation applications. The dielectric function of three-dimensional DSs, also known as bulk Dirac semimetals (BDSs), can be controlled dynamically through a gate voltage that changes their Fermi energy level which results in a metallic (dielectric) response at frequencies below (above) the Fermi energy^74,75^. For example, Cd_3_As_2_, as a three-dimensional (3D) BDS, has attracted research interests due to its chemical stability and extraordinary optical response^74^. It has been fabricated in thin films and at nanoscale through different techniques, including physical vapor deposition (PVD)^76^, pulse laser deposition (PLD)^77^, molecular beam epitaxy (MBE)^78^, chemical vapor deposition (CVD)^79^, and self-selecting vapor growth (SSVG)^80^. The dielectric function of a BDS can be controlled dynamically through altering its Fermi energy by introducing an electric potential difference^77^. For electrically gating and controlling the Fermi energy, the electrolyte gating by means of ion gel could be utilized. The ion gel as a novel material with high conductivity has been used to electrically control the Fermi energy in layers of BDS^78^ and chemical potential of graphene^79^. The properties of the ion gel makes it a suitable alternative for solid polymers and conventional media for electrical conduction applications such as gate material for use in field effect transistors.

In this work, through numerical simulation, we propose GRIN PCs operating as real-time and dynamically tunable flat lenses at THz frequencies. Our GRIN PCs are composed of arrays of BDS pillars in air as the background medium. To the best of our knowledge, this is the first time that BDS-based GRIN PCs are reported. We demonstrate their lensing effect for both transverse electric (TE) and transverse magnetic (TM) polarized incident beams.

## Design and simulation

Permittivity is the determining factor in characterization of the optical properties of BDS. The dielectric function of a BDS is obtained by^74^:

1 $$\varepsilon = \varepsilon_{b} + \frac{{i\sigma_{DS} }}{{\varepsilon_{0} \omega }}$$

in which $\varepsilon_{b}$ is the effective dielectric constant of the background medium, $\varepsilon_{0}$ is the vacuum permittivity, and $\sigma_{DS}$ is the dynamic conductivity of the BDS. The real and imaginary components of $\sigma_{DS}$ are obtained from:

2 $${\text{Re }}\sigma \left( {\Omega } \right) = \frac{{e^{2} }}{\hbar }\frac{{gk_{F} }}{24\pi }{\Omega G}\left( {\frac{\Omega }{2}} \right)$$

3 $${\text{Im }}\sigma \left( {\Omega } \right) = \frac{{e^{2} }}{\hbar }\frac{{gk_{F} }}{{24\pi^{2} }}\left\{ {\frac{4}{{\Omega }}\left[ {1 + \frac{{\pi^{2} }}{3}\left( {\frac{T}{{E_{F} }}} \right)^{2} } \right] + 8{\Omega }\mathop \smallint \limits_{0}^{{\varepsilon_{c} }} \left[ {\frac{{{\text{G}}\left( \varepsilon \right) - {\text{G}}\left( {\frac{{\Omega }}{2}} \right)}}{{{\Omega }^{2} - 4\varepsilon^{2} }}} \right]\varepsilon d\varepsilon } \right\}$$

in which ${\text{G}}\left( E \right) = n\left( { - E} \right) - n\left( E \right) = \sinh \left( {E/T} \right)/[\cosh \left( {E_{F} /T} \right) + \cosh \left( {E/T} \right)]$, $n\left( E \right)$ is the Fermi distribution function, $T$ is the temperature, $E_{F}$ is the Fermi energy, $e$ is the electron charge, $g$ is the degeneracy factor, $\hbar$ is the reduced Planck's constant, $k_{F} = E_{F} /\hbar v_{F}$ is the Fermi momentum, $v_{F} = 10^{6} {\text{ m/s}}$ is the Fermi velocity, $\varepsilon_{c} = E_{c} /E_{F}$, $E_{c}$ is the cutoff energy in which the Dirac's linear spectrum ceases to exist beyond it. $\varepsilon = E/E_{F}$ and ${\Omega } = \hbar \omega /E_{F}$ is the normalized frequency.

In order to design a GRIN PC structure, the photonic Band structure of the PC must be studied. Due to the dispersive nature of the BDS material, we calculated the photonic band structure using the FDTD method via RSoft BandSOLVE simulator. The effective refractive index for lower frequency bands can be calculated using the following equation^57^:

4 $$n_{g} = c\left( {\frac{\partial \omega }{{\partial k}}} \right)^{ - 1}$$

As a result of changes in the band diagram, the effective refractive index of the unit cell can be modified by changing the rods' radii. Therefore, the GRIN PC can be designed by performing a refractive index gradient in an appropriate direction through modifications in unit cell's filling factor.

**Figure 1:**
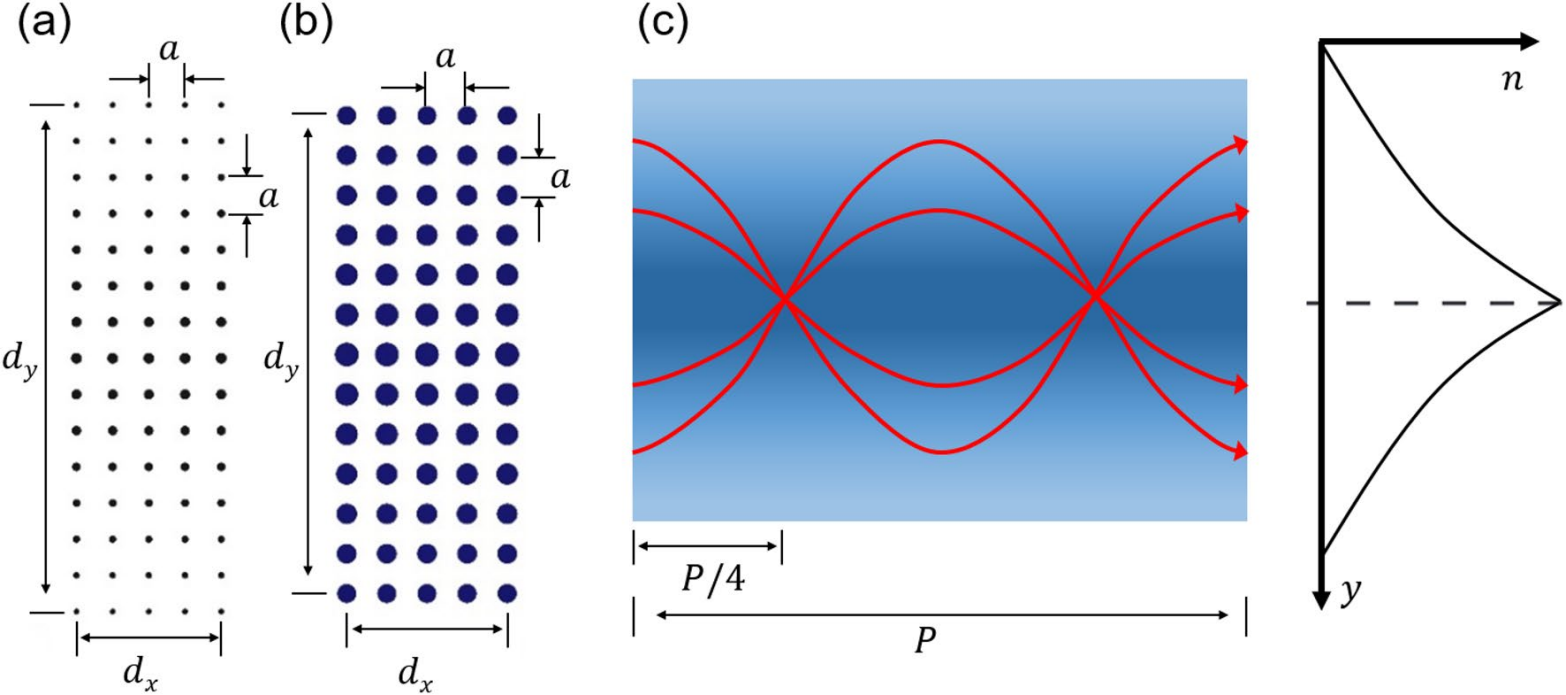
Schematic representation of the proposed GRIN PC composed of BDS rods for (**a**) TM and (**b**) TE polarizations. The structure shown in panel (**a**) is composed of 5 columns and 15 rows. The structure shown in panel (**b**) is composed of 5 columns and 13 rows. The rods’ radii decrease with their distance (above and below along the *y*-axis) from the central axis of the lens. Their radii remain constant along the *x*-axis. The lattice constant, i.e. center-to-center distance between neighboring rods along *x*- and *y*-axis is considered $a = 10 \mu m$. For TM case, the rods’ radii are 1.5 μm, 1.4 μm, 1.3 μm, 1.2 μm, 1.1 μm, 1.0 μm, 0.9 μm, and 0.8 μm as they distance away (above and below) from the central axis along the *y*-direction. Based on $a/\lambda$ = 0.25, the operating central wavelength is 40 μm corresponding to 7.49 THz. For TE case the rods’ radii are 3.0 μm, 2.9 μm, 2.8 μm, 2.7 μm, 2.6 μm, 2.5 μm, and 2.4 μm as they distance away (above and below) from the central axis along the *y*-direction. Based on $a/\lambda$ = 0.27, the operating central wavelength is ~ 37 μm corresponding to 8.09 THz (**c**) Schematic of a GRIN lens demonstrating its pitch, *P*. In order to have focal point outside of the lens, a value of $d_{x} < 0.25P$ is selected. The inset shows a conceptual representation of the refractive index profile along the *y*-axis.

Our proposed two-dimensional GRIN PCs is composed of a square lattice of BDS rods in air background, as schematically shown in Fig. 1. We chose Na_3_Bi or Cd_3_As_2_ as the BDS with $\varepsilon_{b} = 12$ and $g = 4$. The proposed GRIN medium can be fabricated experimentally through electron beam lithography^81^. In order to investigate the focusing properties of the designed 2D GRIN PCs for TM and TE polarizations, respectively, the dimensions were selected as $\left( {d_{x} ,d_{y} } \right) = \left( {5a,15a} \right)$ and $\left( {5a,13a} \right)$, in which $d_{x}$($d_{y}$) is the dimension of the structure in $x$($y$) direction, where $a = 10\mu m$ is the lattice constant. It should be noted that, for a selected value of $d_{y}$, the oscillation period (pitch, $P$) of the field propagation can be obtained using the time domain simulation for TE (TM) polarized incident beam within the GRIN PC. Knowing the pitch enables us to obtain the focusing, collimation and diverging effects by carefully choosing the length of the GRIN PC^82^. In order to have focal point outside of the lens, a value of $d_{x} < 0.25P$ is selected, in our case $d_{x}$ ~ 50 μm.

Numerical analysis was performed through the 2D finite-difference time-domain (FDTD) method using Lumerical software. The simulations were performed with ${\Delta }x = {\Delta }y = a/100$ mesh sizes for adequate accuracy. The simulation domain is covered with perfectly-matched layers (PML) boundaries to absorb the outgoing light. The structure is illuminated with a Gaussian source propagating from left to right.

## Results and discussions

**Figure 2:**
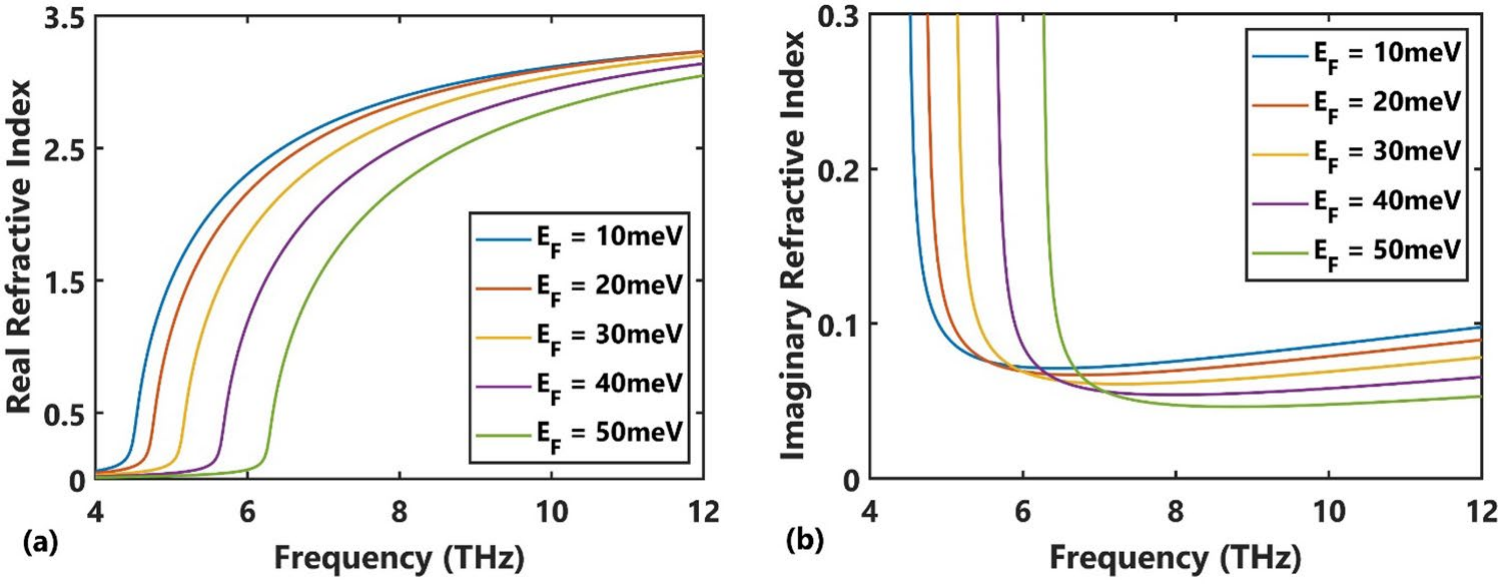
(**a**) Real and (**b**) imaginary refractive index of the BDS material vs. frequency at different Fermi energies.

We considered Fermi energies ($E_{F}$) of 10, 20, 30, 40, and 50 meV. The real and imaginary parts of the permittivity of the selected BDS, calculated at room temperature ($T$ = 300 K) using Eq. (2) and Eq. (3) is shown in Fig. 2(a,b), respectively, as a function of the frequency (4–12 THz) at different Fermi energies. A gradual increase in the real part of the refractive index is observed up to certain frequencies, followed by a sharp increase at higher frequencies. However, a sudden drop in the imaginary part of the refractive index was noted up to certain frequencies followed by a gradual increase. Due to the relatively low imaginary refractive index at frequencies greater than $f = 7{\text{ THz}}$, the absorption associated with the chosen BDS is relatively low at the selected Fermi energies.

**Figure 3:**
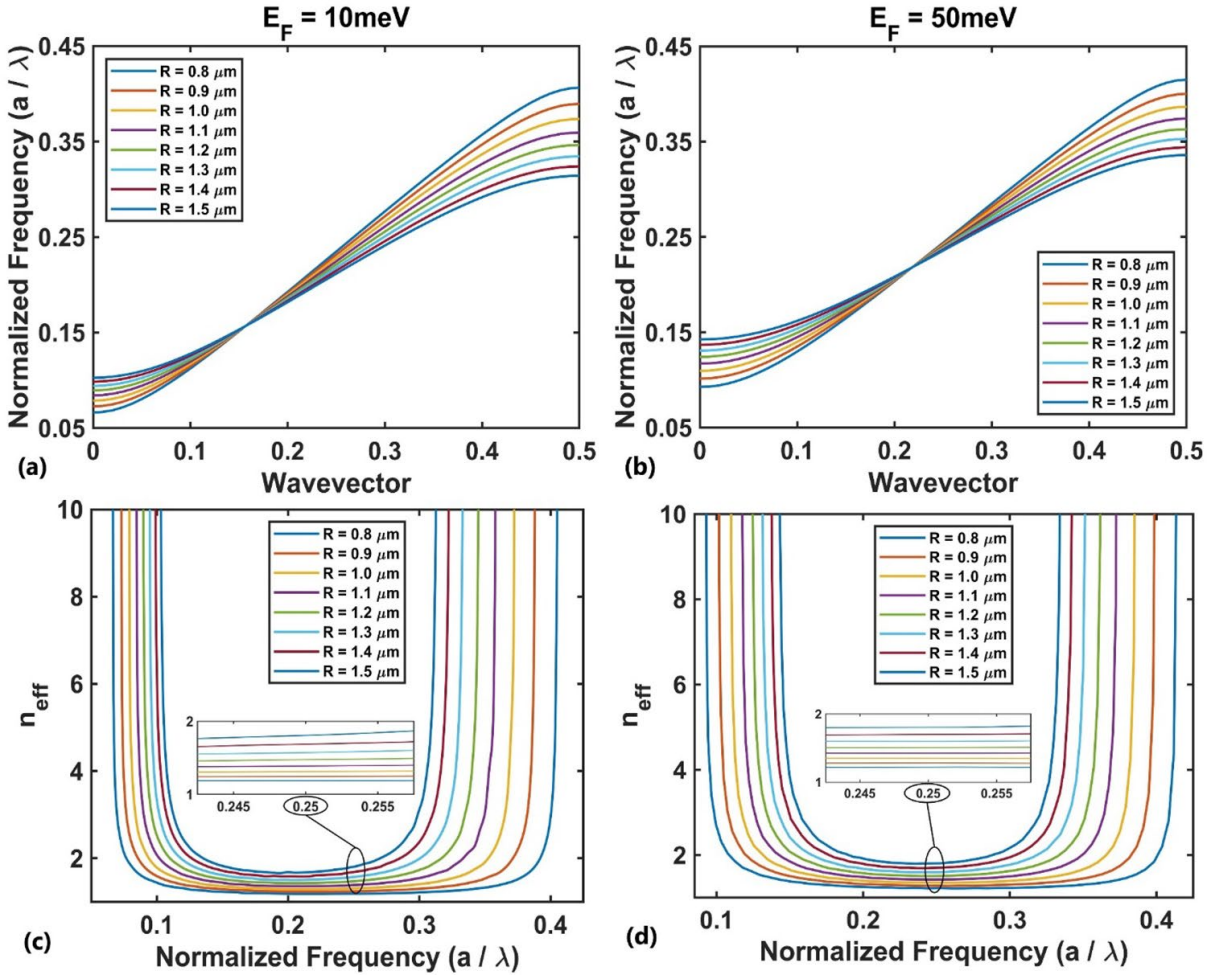
The first TM photonic band dispersion diagram at the Fermi energies of (**a**) $E_{F} = 10{\text{meV}}$ and (**b**) $E_{F} = 50{\text{meV}}$ for variations in the rod's radius, and the corresponding effective refractive index at (**c**) $E_{F} = 10{\text{meV}}$ and (**d**) $E_{F} = 50{\text{meV}}$.

For TM polarization, we consider the first TM band because its slope is nearly constant and the effective refractive index can be readily calculated using the Eq. (4) which has superiority over the effective medium theory method^83^. Figure 3(a,b) represent the radius-dependent dispersion diagram (normalized frequency $a/\lambda$ vs. wavevector) for the first TM band for rods’ radii of $0.8 \mu m$ to $1.5 \mu m$ for the Fermi energy of $E_{F} = 10{\text{ meV}}$ and $E_{F} = 50{\text{ meV}}$, respectively. These range of rods’ radii would provide a desired gradient of refractive index along the *y*-direction. The corresponding effective refractive index of the bands for different values of rods’ radii are shown in Fig. 3(c,d) at two Fermi energies $E_{F} = 10{\text{meV}}$ and $E_{F} = 50{\text{meV}}$, respectively. The presented diagrams appear nearly flat over a specific normalized frequency range (~ 0.22–0.27 corresponding to ~ 6.6–8 THz), and the width of the flat region decreases as the radius of the rod increases. Furthermore, as the radius of the rod increases, the effective refractive index increases for a fixed value of the normalized frequency within the flat region. Therefore, it is possible to find the effective refractive index gradient by reducing the radius of the BDS rods in a particular direction. The produced gradient has the capability of focusing the incident plane wave with a normalized frequency within the flat region.

**Figure 4:**
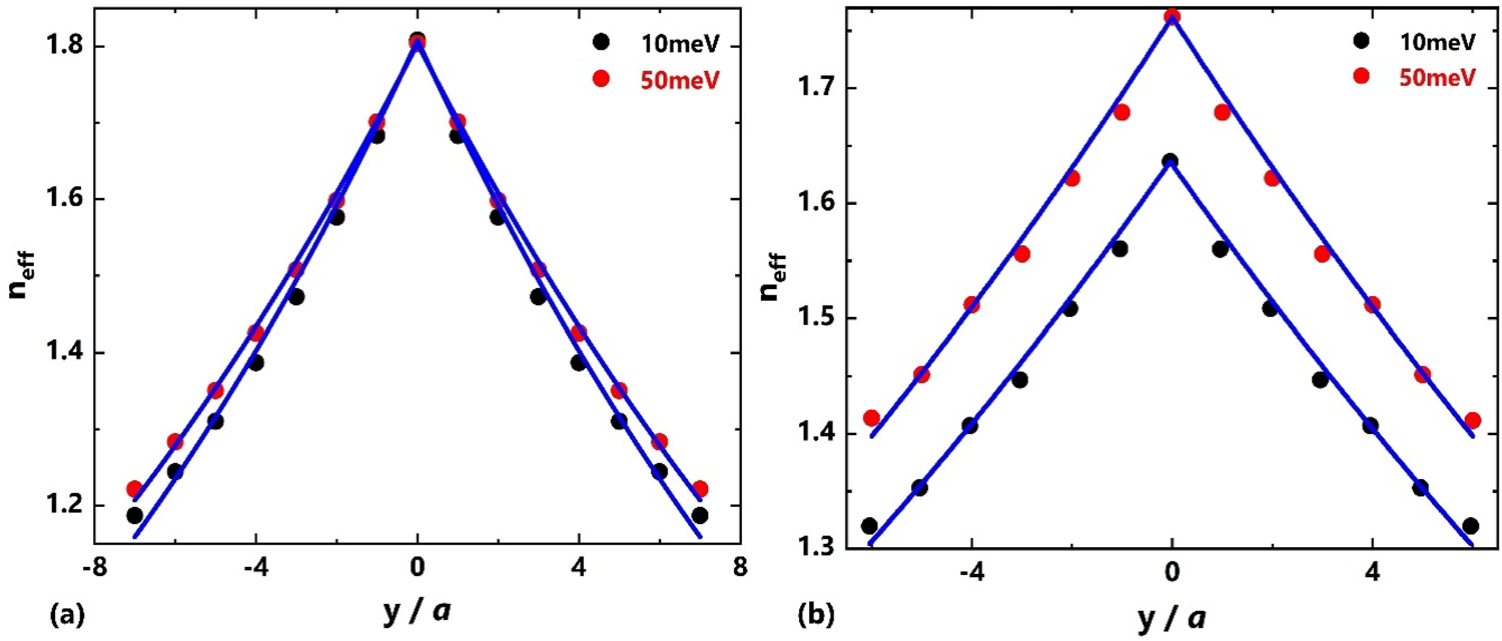
The effective refractive index profile at (**a**) normalized frequency of $a/\lambda = 0.25$ for TM polarization and (**b**) normalized frequency of $a/\lambda = 0.27$ for TE polarization at two Fermi energies $E_{F} = 10{ }meV$ and $E_{F} = 50{ }meV$.

Figure 4a shows the distribution of the effective refractive index along the transverse *y*-direction, obtained from the data presented in Fig. 3(c,d), for normalized frequency of $a/\lambda = 0.25$ and Fermi energies $E_{F} = 10{ }meV$ and $E_{F} = 50{ }meV$, which are fitted to an exponential function $n_{eff} \left( y \right) = n_{0} exp^{ - \beta \left| y \right|}$ (solid curve) with $\beta = 0.0635a^{ - 1}$ (gradient factor) and $n_{0} = 1.808$ (the effective index in the center of the structure) for $E_{F} = 10{ }meV$, and $\beta = 0.0574a^{ - 1}$ and $n_{0} = 1.804$ for $E_{F} = 50{ }meV$. It can be seen that the gradient factor, as a result, the focusing strength of the designed GRIN medium is influenced by the changes in Fermi energy for TM polarization and therefore its optical properties can be controlled through changing Fermi energy. There is a relation between pitch ($P$) and gradient factor, i.e., $P.\beta = 2\pi$^37^, where by increasing the vertical size, $d_{y}$, the gradient factor decreases and the pitch increases. Therefore, the focusing ability of the GRIN medium weakens.

**Figure 5:**
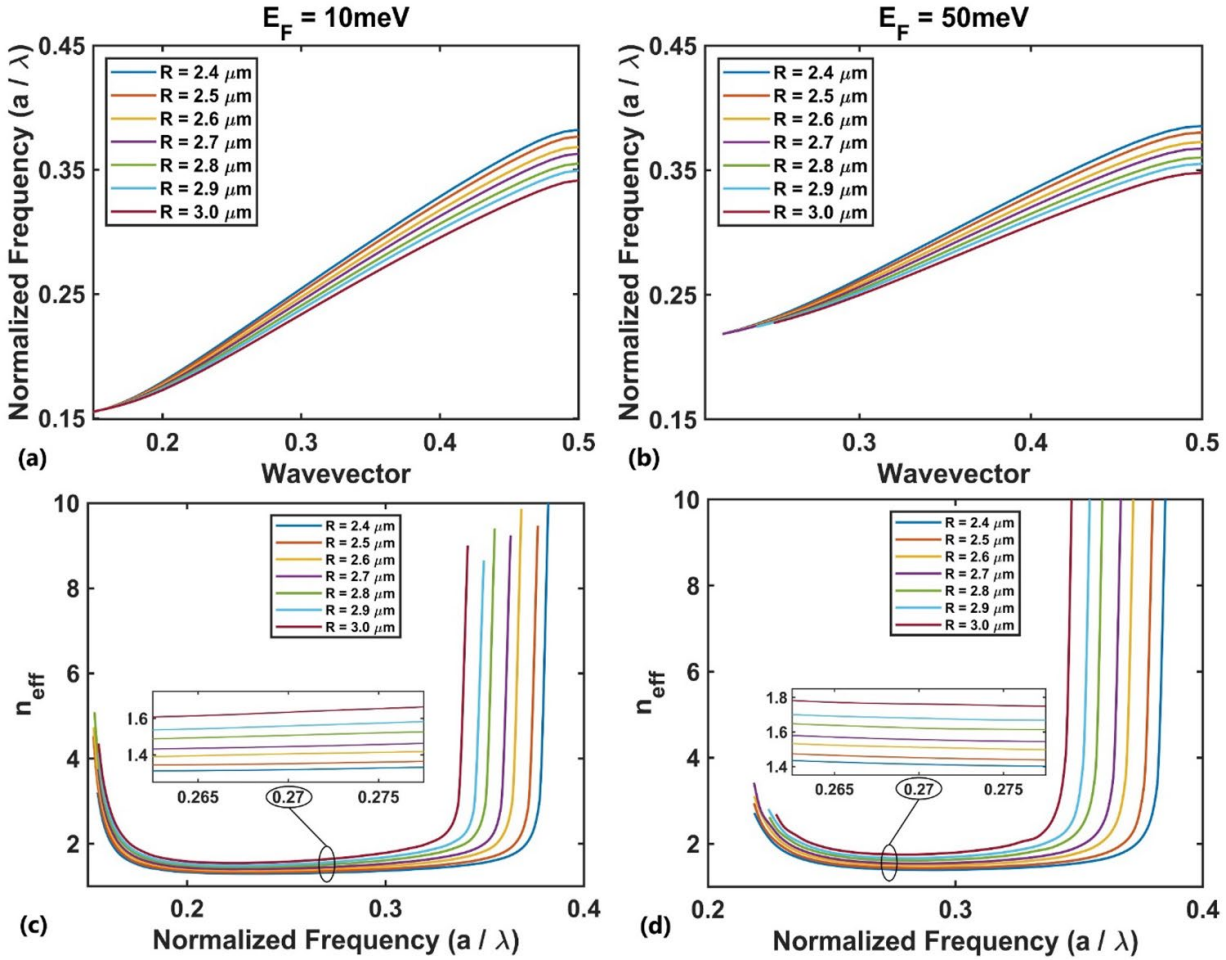
The second TE photonic band's dispersion diagram at the Fermi energies of (**a**) $E_{F} = 10{\text{meV}}$ and (**b**) $E_{F} = 50{\text{meV}}$ for variations in the rod's radius, and the corresponding effective refractive index at (**c**) $E_{F} = 10{\text{meV}}$ and (**d**) $E_{F} = 50{\text{meV}}$.

For TE polarization, the same processes can be performed, where the TE photonic band structure is calculated for different radii from 2.4 μm to 3 μm. Considering $a$ = 10 μm, the numerical results show that the first TE band is located in the frequency range lower than 7 THz, where the absorption of the BDS material is remarkable due to its high imaginary refractive index (Fig. 2b). So, the second TE band diagram was chosen, as shown in Fig. 5(a,b) for Fermi energies $E_{F} = 10{\text{ meV}}$ and $E_{F} = 50{\text{meV}}$, respectively. The corresponding effective refractive index values are shown in Fig. 5(c,d). The presented diagrams appear nearly flat over a specific normalized frequency range (~ 0.25–0.3 corresponding to 7.5–9 THz), and the width of the flat region decreases as the radius of the rod increases. The overlapping operational frequencies of the design examples shown here for both TM and TE polarizations is 7.5–8 THz.

Similar to TM polarization, the effective refractive index gradient can be obtained by reducing the radius of rods along the transverse *y*-direction. As shown in Fig. 4b, the obtained refractive index values for normalized frequency of $a/\lambda = 0.27$ are fitted to an exponential function $n_{eff} \left( y \right) = n_{0} exp^{ - \beta \left| y \right|}$ with $\beta = 0.0378a^{ - 1}$ and $n_{0} = 1.635$ at $E_{F} = 10meV$, and $\beta = 0.0385a^{ - 1}$ and $n_{0} = 1.762$ at $E_{F} = 50meV$. It is clear that, as the Fermi energy increases the effective refractive index profile shifts towards higher values with a little change in gradient factor. Therefore, the tunability of the optical properties of the proposed lens will not be significant compared to TM polarization.

**Figure 6:**
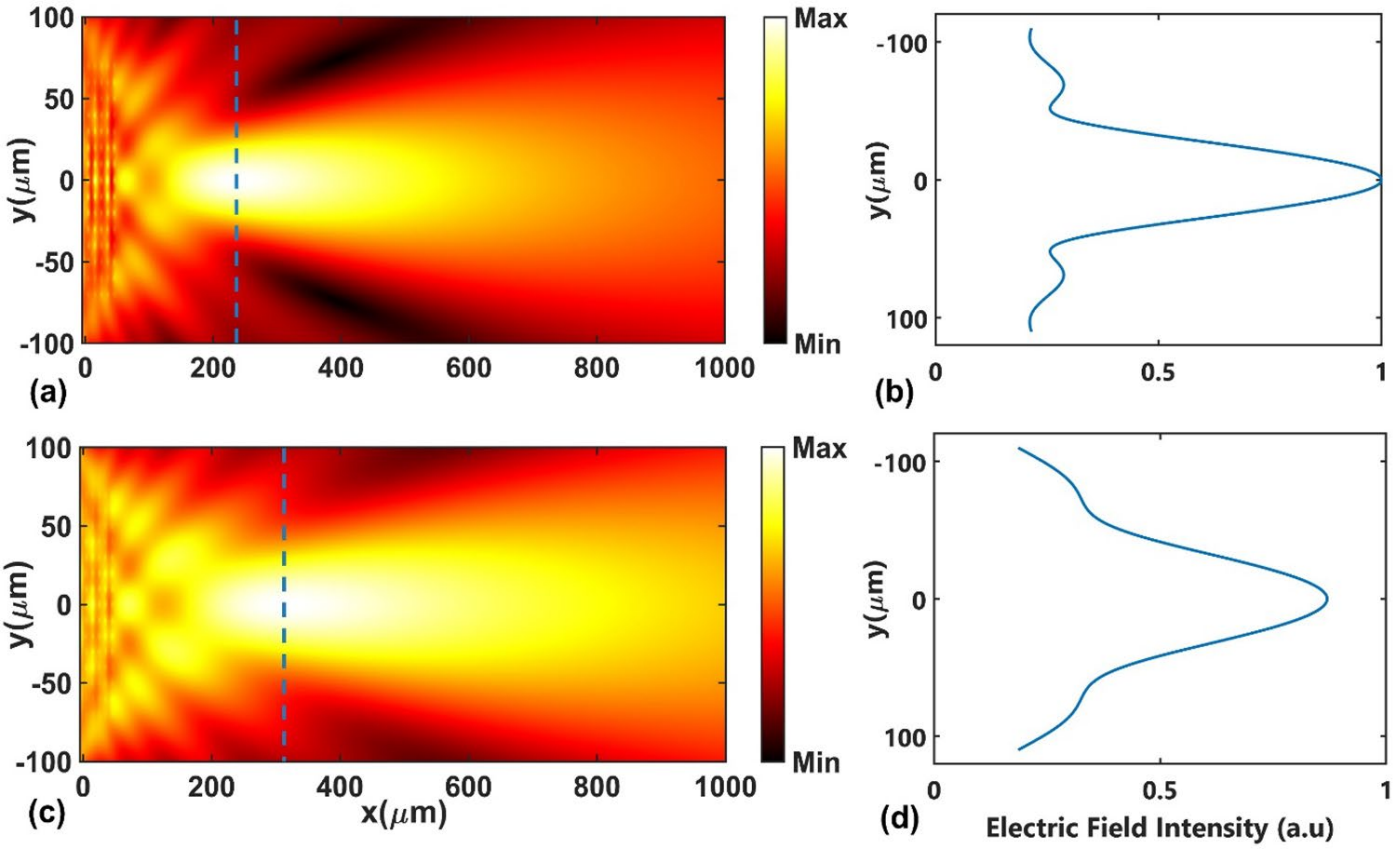
(**a**, **c**) The electric field intensity distribution of TM polarization at normalized frequency of $a/\lambda =$ 0.25 and (**b**, **d**) the transverse profile of the electric field intensity at the focal point at Fermi energies $E_{F} = 10$ meV and $E_{F} = 50$ meV, respectively.

The focusing properties of the proposed GRIN PC were studied at the designed frequency of $a/\lambda = 0.25$ located at first TM band, in which a linear relationship exists between the frequency and the wave vector (i.e., the flat region in the diagram of the effective refractive index vs. normalized frequency). The simulation result for electric field intensity distribution is shown in Fig. 6a for Fermi energy $E_{F} = 10$ meV. According to the obtained results and numerical simulations, it is clear that the designed GRIN PC acts as a lens and focuses the incoming light at a certain focal point. The principle for focusing of light by a flat GRIN PC lens is similar to the focusing principle in bulk gradient index lenses^84^; as light gradually undergoes refraction and reflection during its propagation in a GRIN medium, its trajectory follows a curve path, depending on the index profile within the medium, that can lead to collimation, divergence, or focusing of the incident light^82^. The transverse profile of the electric field intensity at the focal point is shown in Fig. 6b. The focal distance (FD) of the lens, defined as the distance from the exit face to the focal point, could be controlled by changing the Fermi energy, since the BDS's dielectric function can be modified by changing the Fermi energy. As it is clear from Fig. 6c, which is the simulation results for the same incident normalized frequency at Fermi energy $E_{F} =$ 50 meV, the FD of the structure has been increased. Figure 6d demonstrates the transverse profile of the normalized intensity at the focal point of the lens at $E_{F} = 50$ meV.

**Figure 7:**
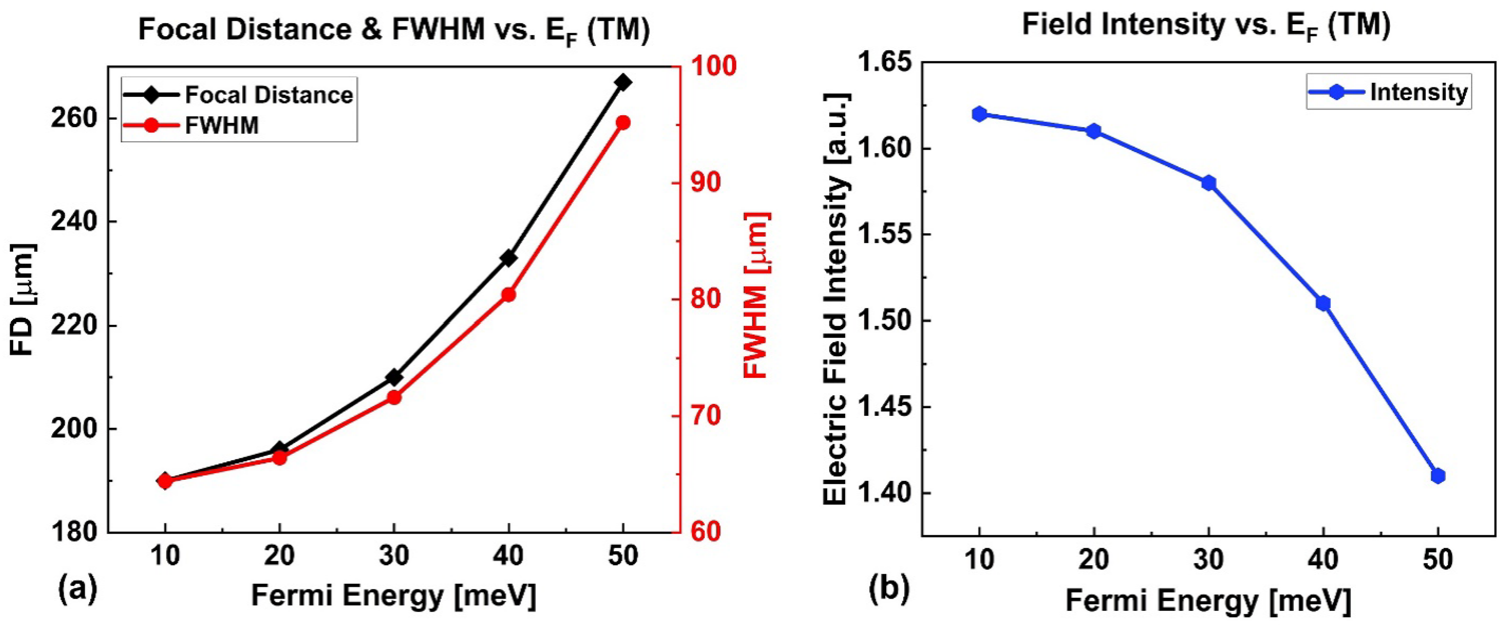
(**a**) Focal distance and FWHM and (**b**) Intensity vs. Fermi energy for TM polarization.

Figure 7a shows the dependency of the focal distance and FWHM of the focused spot on Fermi energy. Figure 7b shows the behavior of the intensity vs. Fermi energy level in the range of 10–50 meV. It is seen that as the Fermi energy increases, the focal distance increase from 190 μm to 267 μm (77 μm change), and the FWHM increases from 64.4 μm to 95.2 μm (30.8 μm change). But, the intensity of the focused light in the focal point decreases from $1.62{ }a.u.$ to $1.41{ }a.u$ (14% change). A remarkable tunability of about $7.7a$ is achieved for the FD in this case. According to Fig. 4a, as the Fermi energy increases the index modulation ($\Delta n$) decrease along with the gradient factor ($\beta$ = 0.0635 $a^{ - 1}$ at 10 meV vs. $\beta$ = 0.0574 $a^{ - 1}$ at 50 meV). Since $\beta$ represents the depth of index distribution, a smaller $\beta$ indicates a weaker lensing effect. This leads to a weakening of the designed GRIN medium's focusing strength, which leads to a longer FD and larger spot size (i.e. greater FWHM) and lower intensity at the focal point.

**Figure 8:**
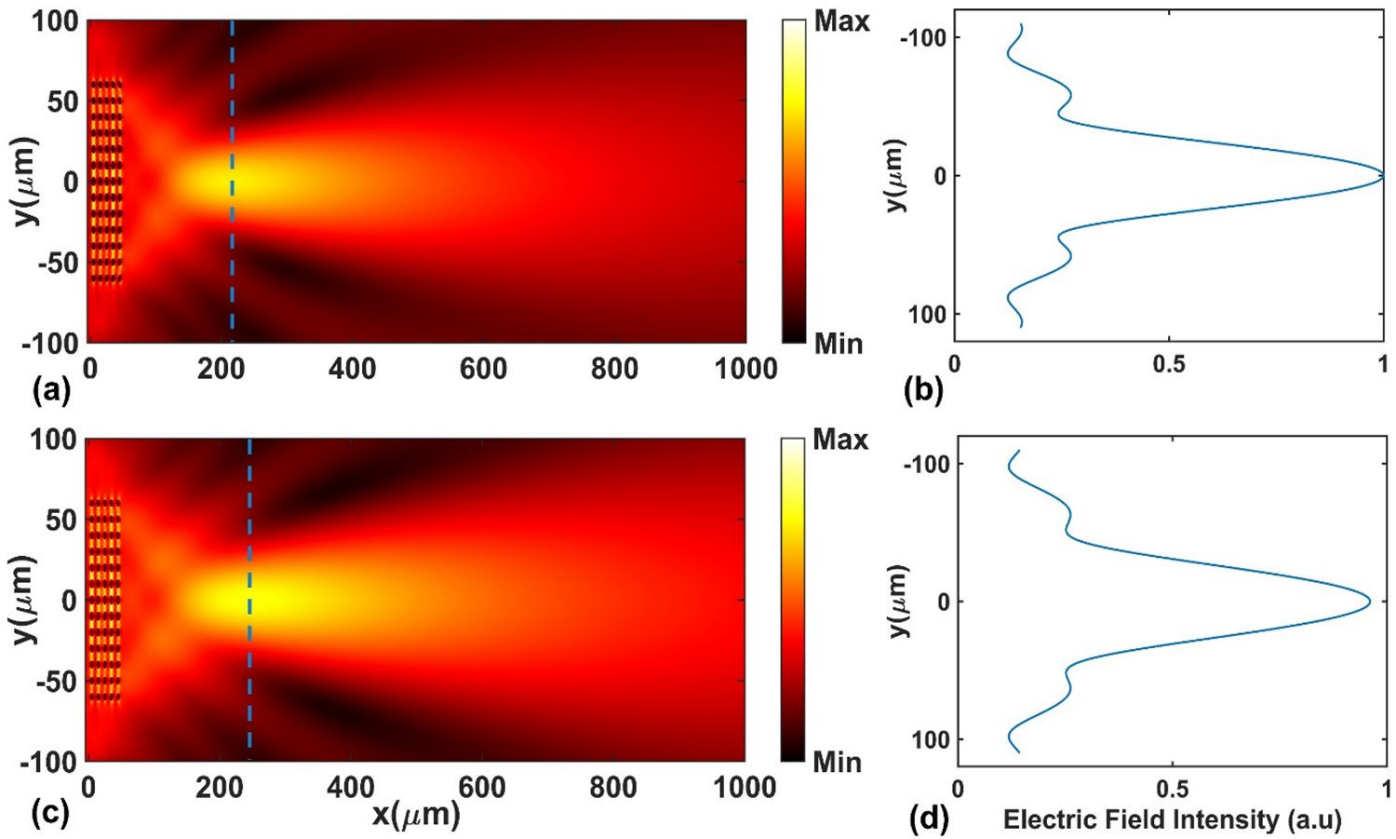
(**a**, **c**) The electric field intensity distribution of TE polarization at normalized frequency of $a/\lambda = 0.27$ and (**b**, **d**) the transverse profile of the electric field intensity at the focal point at Fermi energies $E_{F} = 10meV$ and $E_{F} = 50meV$, respectively.

Next, we investigated the focusing and tuning properties of the designed GRIN medium for incoming normalized frequency of $a/\lambda =$ 0.27 located at the second TE band. Figure 8(a,c) represent the electric field intensity distributions of the incident normalized frequency at two Fermi energies $E_{F} = 10$ meV and $E_{F} = 50$ meV, respectively. It is obvious that the designed GRIN PC acts as a lens and focuses the incident light. Moreover, the changes in Fermi energy affects the focusing properties of the structure. Similar to the TM polarization, we also report the transverse profiles of the normalized intensity at the focal points, as shown in Fig. 8(b and d).

**Figure 9:**
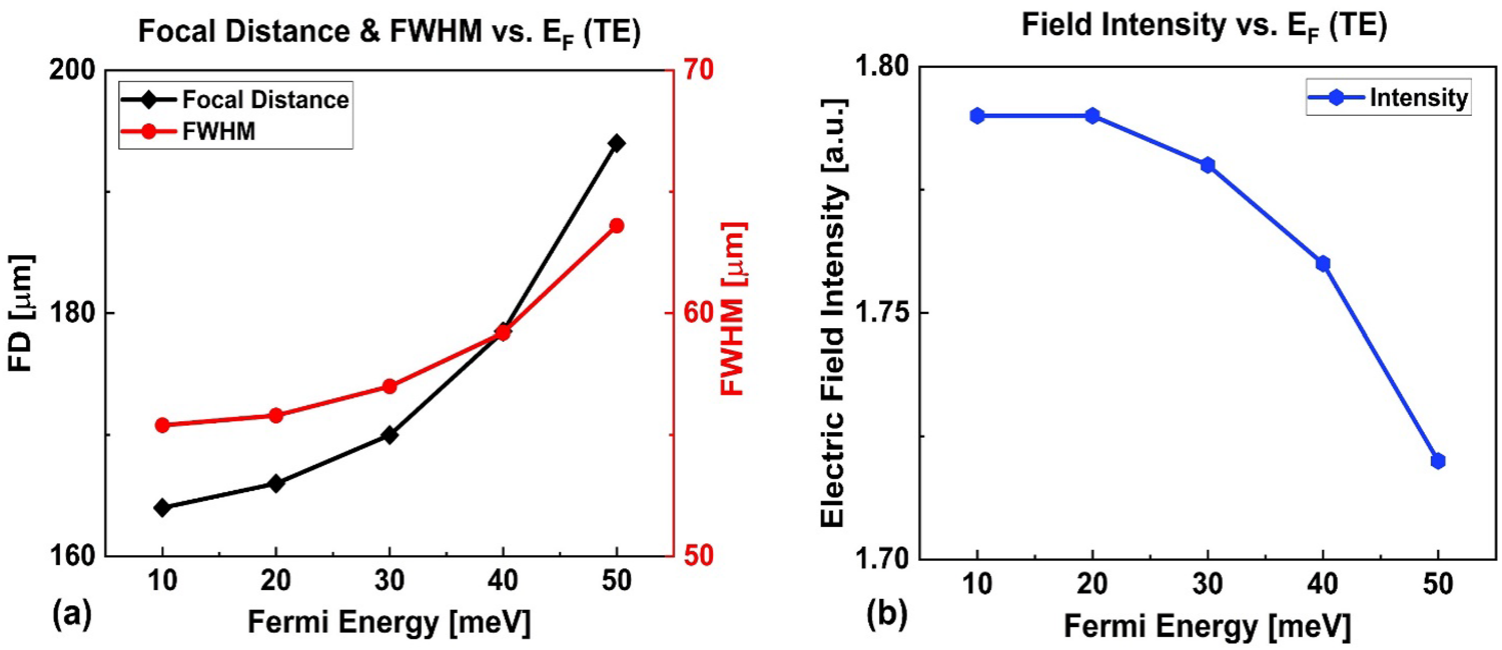
(**a**) Focal distance and FWHM and (**b**) Intensity vs. Fermi energy for TE polarization.

Figure 9a shows the FD and FWHM, and Fig. 9b shows the intensity vs. Fermi energy level in the range of $10 - 50{ }$ meV. It is seen that as the Fermi energy increases, the FD increases from $164$ μm to $194$ μm (30 μm change) and the FWHM increases from 55.4 μm to $63.6$ μm (8.2 μm change). But, the intensity of the focused light in the focal point decreases from $1.79{ }a.u.$ to $1.72{ }a.u.$ (4% change). The tunability of the FD is approximately $3a$ for TE polarization. Comparison between the TM and TE case reveals that the tunability of the FD, FWHM and intensity of the designed GRIN PC for TM polarization is greater than that for the TE polarization. This can be explained by Fig. 4 which shows that when the Fermi energy changes, the variation of the gradient factor ($\beta$) for TM polarization is more remarkable than that for the TE polarization (~ 11% vs. ~ 2%); in the TE case (Fig. 4b), the effective refractive index profile for TE polarization is just shifted towards high values with the increase in Fermi energy, while the $\beta$ changes slightly. Since $\beta$ represents the depth of index distribution, its smaller change based on Fermi energy for TE polarized incident light means that the optical properties of the GRIN PC (FD, FWHM and intensity) are less affected by changing the Fermi energy in that case. It should be mentioned that, in principle, the focusing properties of our proposed lens can be further optimized based on the design goals through optimizing the rods' diameter^22^, their distance in *y*-direction^85^, and the longitudinal dimension of the lens^82^, and the refractive index of the rods.

## Conclusions

We have theoretically designed a tunable flat lens to operate at THz domain based on the concept of GRIN PC. Our proposed structure has a square lattice made of BDS rods with varying radii in air background. It was demonstrated that by changing the Fermi energy of the BDS rods, the effective refractive index within the unit cells can be controlled, hence the focusing properties of these structures. Using this feature, we studied the tuning properties of these structures for TM and TE polarizations of the incident THz beam. It was observed that the tunability of the designed GRIN PC is greater for the TM polarization compared to the TE polarization. In principle, in analogy with GRIN PC lenses, the lensing characteristics of our structures can be further fine-tuned, depending on the design objectives, through manipulation of the rods’ size and separation, and dimensions of the lens.

## Data Availability

The datasets used and/or analyzed during the current study available from the corresponding author on reasonable request.
